# Cysteamine (Lynovex®), a novel mucoactive antimicrobial & antibiofilm agent for the treatment of cystic fibrosis

**DOI:** 10.1186/s13023-014-0189-2

**Published:** 2014-11-30

**Authors:** Cedric Charrier, Catherine Rodger, Jennifer Robertson, Aleksandra Kowalczuk, Nicola Shand, Douglas Fraser-Pitt, Derry Mercer, Deborah O’Neil

**Affiliations:** NovaBiotics Ltd, Cruickshank Building, Craibstone Aberdeen, AB21 9TR UK

**Keywords:** Cystic fibrosis, Mucolytic, Antibacterial, Antimicrobial, Biofilm, Antibiofilm, Synergy

## Abstract

**Background:**

There remains a critical need for more effective, safe, long-term treatments for cystic fibrosis (CF). Any successful therapeutic strategy designed to combat the respiratory pathology of this condition must address the altered lung physiology and recurrent, complex, polymicrobial infections and biofilms that affect the CF pulmonary tract. Cysteamine is a potential solution to these unmet medical needs and is described here for the first time as (Lynovex®) a single therapy with the potential to deliver mucoactive, antibiofilm and antibacterial properties; both in oral and inhaled delivery modes. Cysteamine is already established in clinical practice for an unrelated orphan condition, cystinosis, and is therefore being repurposed (in oral form) for cystic fibrosis from a platform of over twenty years of safety data and clinical experience.

**Methods:**

The antibacterial and antibiofilm attributes of cysteamine were determined against type strain and clinical isolates of CF relevant pathogens using CLSI standard and adapted microbiological methods and a BioFlux microfluidic system. Assays were performed in standard nutrient media conditions, minimal media, to mimic the low metabolic activity of microbes/persister cells in the CF respiratory tract and in artificial sputum medium. *In vivo* antibacterial activity was determined in acute murine lung infection/cysteamine nebulisation models. The mucolytic potential of cysteamine was assessed against DNA and mucin *in vitro* by semi-quantitative macro-rheology. In all cases, the ‘gold standard’ therapeutic agents were employed as control/comparator compounds against which the efficacy of cysteamine was compared.

**Results:**

Cysteamine demonstrated at least comparable mucolytic activity to currently available mucoactive agents. Cysteamine was rapidly bactericidal against both metabolically active and persister cells of *Pseudomonas aeruginosa* and also emerging CF pathogens; its activity was not sensitive to high ionic concentrations characteristic of the CF lung. Cysteamine prevented the formation of, and disrupted established *P. aeruginosa* biofilms. Cysteamine was synergistic with conventional CF antibiotics; reversing antibiotic resistance/insensitivity in CF bacterial pathogens*.*

**Conclusions:**

The novel mucolytic-antimicrobial activity of cysteamine (Lynovex®) provides potential for a much needed new therapeutic strategy in cystic fibrosis. The data we present here provides a platform for cysteamine’s continued investigation as a novel treatment for this poorly served orphan disease.

**Electronic supplementary material:**

The online version of this article (doi:10.1186/s13023-014-0189-2) contains supplementary material, which is available to authorized users.

## Background

Antibiotics, whether administered systemically or by inhalation, remain a mainstay of the cystic fibrosis therapy regimen [1]. Control and at least a degree of resolution of the respiratory infections and bacterial colonisation associated with cystic fibrosis is an essential component of disease management. The altered physiology of the cystic fibrosis respiratory tract makes this almost impossible to achieve. The dehydrated mucus and sputum create an ideal environment for microbial infection and colonisation and a largely impenetrable barrier for access of antibiotics to the bacterial pathogens they are targeting [2].

The co-administration of mucolytic or osmotic agents (e.g. DNAase/pulmozyme®, N-acetylcysteine/Mucomyst® and mannitol/Bronchitol® [3]) to reduce mucus viscosity and elasticity [4] is intended to facilitate increased antibiotic-microbe contact. These therapies can also improve the patient’s ability to expectorate. There is insufficient evidence, however, that these adjunct treatments affect overall antibiotic usage [5]. The antibiotic resistance which this sustained antibiotic regimen inevitably leads to continues to pose an ever-increasing clinical problem in the treatment of cystic fibrosis [6,7]. The need to develop more effective strategies for the resolution of mucus build up and the eradication of the respiratory pathogens that infect and colonise the respiratory tract in cystic fibrosis is acute.

The biofilm mode of growth of *P. aeruginosa* and other bacterial pathogens in the cystic fibrosis lung also leads to higher antibiotic tolerance. This is a further, compounding factor in antimicrobial resistance development in cystic fibrosis and the resulting reduction in effectiveness/life-span of antibiotic treatments [8]. Unresolved infection and acute exacerbations lead to a more rapid deterioration in lung function and increased morbidity and mortality [9]. This cycle can only really be broken by tackling, in parallel, the mucus barrier and eradicating the bacteria it hosts/protects, in both planktonic and biofilm form.

We initially investigated cysteamine as a biofilm and mucus penetration enhancer for improved antibiotic delivery in cystic fibrosis [10,11]. This was on the basis of cysteamine’s previously described ability to selectively disrupt disulphide bonds [12]. Cysteamine was indeed an effective mucolytic and biofilm disrupter/preventer, outperforming currently licensed mucolytic/osmotic agents in these functions. Surprisingly, cysteamine was also directly antimicrobial against *P. aeruginosa*; including mucoid and non-mucoid strains and clinical isolates. Cysteamine was also active against other cystic fibrosis bacterial pathogens. Cysteamine dramatically increased the antimicrobial activity of tobramcyin, ciprofloxacin and colistin; synergising to enhance their potency, increase their spectrum of activity against broader panels of cystic fibrosis relevant bacteria and delivering a marked post-antimicrobial effect.

The intention is to develop cysteamine/Lynovex® for application alongside existing antibiotic therapies. As an adjunct treatment, this may extend the utility and lifespan of conventional antibiotics, whilst at the same time potentially reducing dosing levels and frequency. Of major significance, cysteamine/Lynovex® could help ameliorate or even prevent antimicrobial resistance development if the data we have obtained thus far translates in clinical practice.

Cysteamine is already approved in other non-related clinical indications such as cystinosis and as such, has an established safety profile and has been in clinical use since 1994 [13]. Our own work goes further to demonstrate at least preliminary *in vivo* tolerability and safety following respiratory tract delivery (nebulisation or intratracheal administration) of cysteamine in murine lung infection models. These *in vivo* studies (acute *P.aeruginosa* lung infection models) provided further compelling initial evidence of the antimicrobial activity of cysteamine even when used as a monotherapy in nebulised form against mucoid isolates of *P. aeruginosa*.

The novel properties we describe here for cysteamine are potentially directly applicable to cystic fibrosis and may provide a much needed solution to the urgent need for new therapeutic strategies for this still poorly served, life limiting condition.

## Methods

### Bacterial strains and culture conditions

Bacteria included in this study are listed in Additional file 1: Table S1 and summarised in Table 1. Strains were grown in Mueller-Hinton (MH) broth or agar plates at 37°C. All chemicals and reagents were obtained from Sigma-Aldrich (UK), unless otherwise stated. NH57388A is a mucoid strain of *P. aeruginosa* and NH57388B is a non-mucoid strain of *P. aeruginosa* [14].

**Table 1:** **Minimum inhibitory concentrations and MIC ranges of cystic fibrosis respiratory pathogens versus cysteamine**

**Bacteria (n = 103)**	**Median MIC**_**100**_ **(μg/ml)**	**Range (μg/ml)**
*P. aeruginosa* (n = 42)	250	250-500
*P. fluorescens* (n = 2)	250-500	250-500
*P. putida* (n = 1)	250	NA
*B. cepacia* (n = 3)	250	250-500
*B. cenocepacia* (n = 3)	500	500-1000
*B. multivorans* (n = 3)	500	500
*Ach. xylosidans* (n = 7)	500	250-500
*Ach. denitrificans* (n = 1)	500	NA
*Al. faecalis* (n = 2)	250-500	250-500
*C. indologenes* (n = 2)	250-500	
*K. pneumoniae* (n = 3)	250	250-1000
*Pan. apista* (n = 1)	500	NA
*Pan. pnomenusa* (n = 1)	1000	NA
*R. pickettii* (n = 3)	500	500-1000
*R. mannitolilytica* (n = 1)	500	NA
*Sph. paucimobilis* (n = 2)	1000	1000
*St. maltophilia* (n = 7)	500	250-1000
*S. aureus* (n = 14)	250	250-500

Data are median MIC_100_ of triplicate samples in experiments repeated 3 times. P = Pseudomonas, B = Burkholderia, Ach = Achromobacter, Al = Alcaligenes, C = Chryseomonas, K = Klebsiella, Pan = Pandoraea, R = Ralstonia, Sph = Sphingomonas, St = Stenotrophomonas, S = Staphylococcus, NA = Not applicable.

### Antibiofilm activity

The effect of cysteamine and other mucolytic and antibiotic agents on biofilm prevention of *P. aeruginosa* PAO1 were visualized using a Bioflux microfluidic system (Fluxion, USA) and transmitted light microscopy (Axiovert 40, Carl Zeiss, UK). Compounds tested included cysteamine and other mucolytic agents (N-acetylcysteine, DNase I, alginate lyase) and tobramycin.

Outlet wells of 48-well microfluidic flow cell plates (Fluxion, USA) were primed with 0.5 × MH broth for 1 min and then inoculated with 200 μl of a mid-late exponential phase culture of *P. aeruginosa* PAO1 (OD_625_ ~ 0.7) in MH broth for 5 s at 1 Dyn/cm^2^ via the outlet wells. The flow cell plates were then incubated statically at 37°C for 45 min to allow the bacteria to adhere to the glass flow cells. Images were captured from all experimental capillaries (× 40 magnification) at this time point (t = 0 h). The compound of interest was solubilised in MH broth and introduced into the inlet well. Flow was established at 0.5 Dyn/cm^2^ at incubation at 37°C for 16 h was initiated. Two specific adjacent capillaries were selected and photographed every 15 min for the 16 h incubation to create an .avi movie. At t = 16 h, all capillaries were again photographed at × 40 magnification. All images were captured using a QImaging QICAM camera and analysed using QCapture Suite software (QImaging, Canada). All experiments were carried out at least 3 times, and presented images and movies are representative of a single experiment.

To determine whether cysteamine, tobramycin or combinations thereof elicited a post-antimicrobial effect, at the conclusion of biofilm prevention experiments, 10 μl samples of cells were removed from each outlet well and added to 190 μl MH broth and incubated in a 96-well microplate for 24 h at 37°C, with no added antimicrobials, and recovery of growth was monitored by taking hourly absorbance readings (625 nm) using a BioTek Synergy microplate reader (BioTek, USA).

The minimum bacterial eradication concentration (MBEC) was determined against biofilms of *P. aeruginosa* ATCC27853 and *P. aeruginosa* DSMZ1299 using the method of O’Neill, et al. [15] with the following modifications. Isolates were cultured for 24 h at 37°C in 5 ml of MH broth. Cultures were diluted to the 0.5 McFarland Standard in MH broth and 200 μl aliquots were added to each well of a 96-well tissue culture-treated polystyrene plate. After 24 h growth at 37°C, the plates were washed three times with phosphate-buffered saline (PBS) to remove any unattached bacteria and treated with test compound for 24 h. Following this second incubation plates were washed three times with PBS to remove any unattached bacteria then dried for 1 h at 60°C prior to staining with 0.4% crystal violet solution. The optical density (492 nm) was used as an index of bacterial adherence to the surface and biofilm formation. Experiments were performed in triplicate, the results were averaged, and standard errors were calculated. To compensate for background absorbance, OD readings of the sterile medium with both the fixative and dye were averaged and subtracted from all of the experimental values. A biofilm-positive phenotype was defined as OD ≥ 0.2 at 492 nm.

### Mucolytic activity

The macrorheological impact of cysteamine on individual mucus components was determined by quantifying changes in viscoelasticity of a sterile 20% (w/v) solution of porcine mucin solution or a sterile 5 mg/ml calf thymus DNA solution following exposure to cysteamine versus other, control mucolytic agents [16]. Mucolytic treatments were prepared at 10 mg/ml and exposed to 20% (w/v) mucin solution for 16 h at 37°C. For DNA, cysteamine was tested at concentrations of 1 mg/ml and 125 μg/ml and recombinant human DNAse I (rhDNase I) was tested at 10U/ml. In both experiments, an equivalent volume of water (4 μl) was used as a control for possible dilution effects. Changes in mucin and DNA viscoelasticity were determined by distance travelled (mm) as a function of time (min) through sterile 1 ml serological pipettes (Greiner Bio-One; 2.77 mm internal bore) at room temperature.

### Effect of cysteamine on mucin production by normal human bronchial epithelial (NHBE) cells

NHBE cells (Lonza Group Ltd, UK) were cultured according to the suppliers’ instructions. Cells were transferred from their growth media to Clonetics B-ALI air-liquid interface medium (Lonza Group Ltd, UK) (basal aspect only) and to collagen-coated 24-well transwells at a density of 5 × 10^4^ cells per insert. Fully differentiated monolayer cultures were generated per supplier’s instructions. Transwells were then treated with 1 mg/ml cysteamine (added to the basal layer medium) or with B-ALI media only (untreated controls) for 7 d, with media/treatment changed in all cultures every 48 h. Free apical fluid generated by each of the monolayers was collected and its volume measured. Levels of apical mucin generation were then assessed in all cultures by a method based on that of Handra-Luca and co-workers [17]; transwell filters and cells were fixed with 10% (v/v) formalin, pH 7.0 for 24 h, rinsed with sterile PBS and stained with Alcian blue (1% in 3% (v/v) acetic acid, pH 2.5 for 15 min, photographed for assessment of gross staining levels and monolayers examined microscopically.

### Antibacterial susceptibility testing

Minimum Inhibitory Concentrations (MICs) were determined by the broth microdilution procedure described in Clinical and Laboratory Standards Institute (CLSI) approved standard M07-A9 [18]. Additionally, MICs were determined as described above, but in artificial sputum medium [19] and M9 minimal medium [20]. The MIC_100_ of cysteamine and other test item antimicrobials employed in this study was determined as the lowest concentration of antimicrobial showing total inhibition of bacterial growth.

### Determination of the effect of combinations of cysteamine and antibiotics versus *P. aeruginosa* PAO1

To determine whether the interaction of cysteamine with the antibiotics tobramycin, colistin, ciprofloxacin and gentamicin was synergistic, additive, antagonistic or there was no effect the Fractional Inhibitory Concentration (FIC) was determined after carrying out checkerboard MIC testing [21]. For all of the wells of the microdilution plates that corresponded to an MIC, the sum of the FICs (ΣFIC) was calculated for each well with the equation ∑FIC = FICA + FICB = (CA/MICA) + (CB/MICB), where MICA and MICB are the MICs of drugs A and B alone, respectively, and CA and CB are the concentrations of the drugs in combination, respectively.

### *In vivo* efficacy & tolerability

Cysteamine was administered directly into the lungs of mice (post-infection with *P. aeruginosa*) either by intratracheal delivery or nebulisation in murine models of respiratory infection by Ricerca Biosciences LLC (Taiwan) and Euprotec Ltd (UK), respectively.

Briefly, in the first study (Ricerca), groups of 5 ICR male mice were immunosuppressed prior to intranasal infection (3–10 × 10^4^ CFU/mouse *P. aeruginosa* ATCC27853). Cysteamine (5 mg/kg) or tobramycin (3 mg/kg) were then administered intratracheally (IT) in 2 doses, 10 min and 6 h following infection In the second study (Euprotec), groups of 8 immunosuppressed ICR mice were infected with *P. aeruginosa* EUPPA103 (6.5 × 10^4^ CFU/mouse by intranasal injection). Mice were nebulised 1 h post-infection with aqueous cysteamine (one dose of 4.2 mg/ml for 5, 10 or 20 min) or aqueous tobramycin (4.2 mg/ml for 10 min).

Endpoints of both studies were assessment of lung tissue bacterial burden 25 h post-infection. Statistical analyses were performed for the second study using StatsDirect - Kruskal-Wallis: all pairwise comparisons (Conover-Inman) for vehicle versus treatment group comparison. The lower limit of detection was approximately 50 CFU/g of lung tissue.

## Results and discussion

### Antibiofilm activity of cysteamine

**Figure 1:**
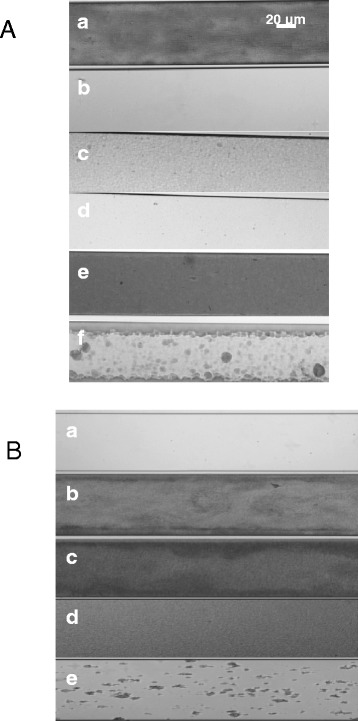
**Antibiofilm activity against** ***P. aeruginosa*** **PAO1 Biofilms of A – cysteamine, N-Acetylcysteine, rhDNase and alginate lyase and B – cysteamine and cysteamine in combination with tobramycin. A**- a: Untreated control; b: 1 mg/ml Cysteamine; c: 1 mg/ml N-acetylcysteine; d: 1 mg/ml Cysteamine hydrochloride; e: 1 mg/ml rhDNase I; f: 1 mg/ml Alginate lyase. **B**- a: Control 0 h; b: Control 16 h; c: 100 μg/ml cysteamine; d: 10 μg/ml tobramycin; e: 100 μg/ml cysteamine & 10 μg/ml tobramycin. In all cases, *P. aeruginosa* PAO1 biofilms were seeded, and their growth monitored, in the presence of the mucoactive and/or antibacterial compounds listed above for 16 h in the BioFlux200 microfluidic system at a flow rate of 0.5 Dyn/cm^2^.

The ability of cysteamine and cysteamine hydrochloride to prevent formation of *P. aeruginosa* PAO1 biofilms was compared with other mucoactive compounds currently used in clinical practice, or in development as a treatment for CF (rhDNAse I, alginate lyase and N-acetylecysteine) was determined using the Bioflux 200 microfluidic system. Cysteamine and cysteamine hydrochloride were effective in preventing *P. aeruginosa* biofilm formation (Figure 1A-b, 1A-d), as no microbial growth is seen, and superior to the other mucolytic agents tested; N-acetylcysteine (Figure 1A-c), rhDNase I (Figure 1A-e) and alginate lyase (Figure 1A-f), where biofilm formation is clearly evident, as also seen in the untreated control (Figure 1A-a).

The biofilm prevention potential of cysteamine was then investigated in the presence of one of the cystic fibrosis ‘gold standard’ antibiotics, tobramycin. A synergistic trend was observed (Figure 1B-e) in that tobramycin potentiated the antibiofilm activity of cysteamine and *vice versa* whereas alone, neither cysteamine (Figure 1B-c) nor tobramycin (F1B-d) prevented significant biofilm formation. This is especially interesting as the concentration of cysteamine used was subsequently determined to be 2.5-times lower than the MIC_100_ of cysteamine versus *P. aeruginosa* PAO1, although the MIC_100_ of this strain to tobramycin was 1 μg/ml. It should be pointed out that the biofilm inoculum was a much more cell-dense, exponential growth phase culture of *P. aeruginosa* PAO1 (OD_625_ ~ 0.7), not the usually applied CLSI inoculum of ~5 × 10^5^ cfu/ml (OD_625_ < 0.1) [18], so both putative antibiofilm agents were being tested at levels significantly below their MIC_100_ value. Therefore, a sub-MIC concentration of cysteamine acted synergistically with tobramycin to prevent biofilm formation. This was our first evidence of cysteamine having a direct antimicrobial effect against *P. aeruginosa* versus it being solely antibiofilm in function. Moreover, when comparing Figure 1A-c (N-acetylcysteine) and 1A-e (rhDNase I) to Figure 1B-e, the combination of cysteamine and tobramycin was more potent in biofilm prevention than existing clinical muco-active used for CF treatment.

**Figure 2:**
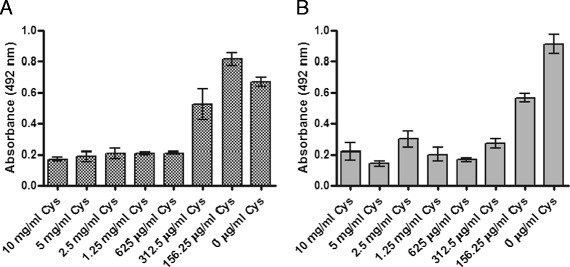
**Determination of the minimum biofilm eradication concentration of cysteamine against established** ***P. aeruginosa*** **DSMZ1299 (A) and** ***P. aeruginosa*** **ATCC27853 (B) biofilms.** Cys – cysteamine. The optical density (492 nm) of crystal violet released from adherent cells within 24 h biofilms was used as an index of biofilm formation. A biofilm-positive phenotype was defined as OD ≥ 0.2. To compensate for background absorbance, OD readings of the sterile medium with both the fixative and dye were subtracted from all the experimental values. Experiments were performed in triplicate and results are presented as means. Error bars represent the standard error of the mean.

Prevention of *P. aeruginosa* and other bacterial biofilms is a highly desirable characteristic for any new CF candidate therapy, but in the clinical setting, a truly effective CF treatment targeted to the chronic and recurrent respiratory infections associated with this condition should also be able to disrupt and even eradicate bacteria growing in established biofilms. As such, the biofilm eradication potential of cysteamine was assessed by determining its Minimum Biofilm Eradication Concentration (MBEC) against two *P. aeruginosa* biofilms (2a – DSMZ1299 & 2b – ATCC27853). As shown in Figure 2A-B, cysteamine was able to eradicate *P. aeruginosa* biofilms with an MBEC of 625 μg/ml (~2-3 times the MIC_100_; Table 1).

### Mucolytic activity of Cysteamine versus Mucin and DNA

**Figure 3:**
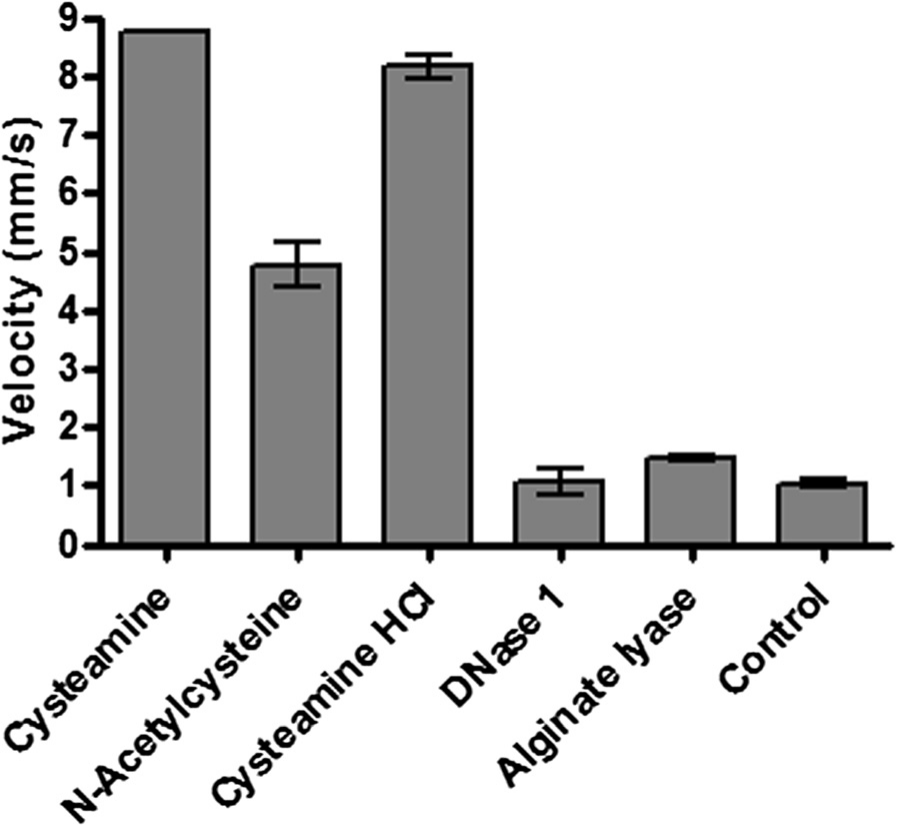
**Positive macro-rheologic impact of cysteamine, N-Acetylcysteine, rhDNase and alginate lyase on mucin.** 20% (w/v) porcine stomach mucin was exposed to the 10 mg/ml of the cysteamine and the mucoactive agents listed above for 24 h at 37°C and the viscosity of the samples determined by measuring velocity (distance moved (mm) over time (s)). An equal volume of distilled water (4 μl) was used as a control. The experiment was carried out in triplicate and the bars represent the mean.

**Figure 4:**
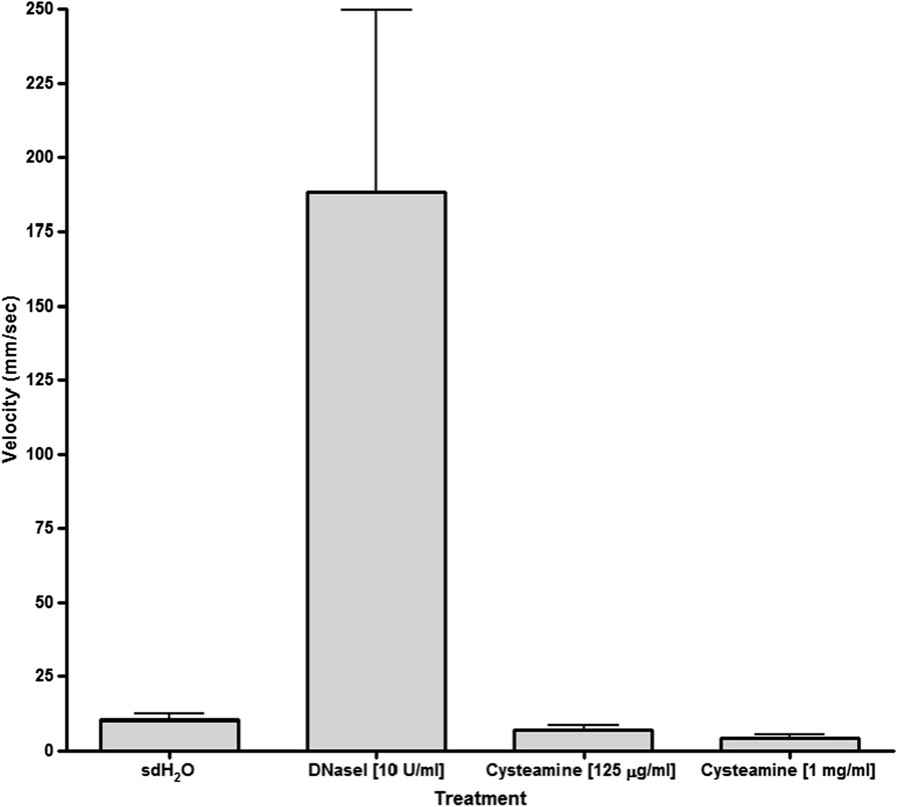
**Neutral macro-rheologic impact of cysteamine and rhDNase on DNA.** 5 mg/ml calf thymus DNA was exposed to 1 mg/ml or 125 μg/ml of cysteamine and 10 U/ml rhDNase I for 2 h at 37°C and the viscosity of the samples determined by measuring velocity (distance moved (mm) over time (s)) DNase – rhDNAse I; Cys – cysteamine. The experiment was carried out in triplicate and the bars represent the mean.

Cysteamine has already been described as disulfide bond disrupter [12]. This, plus its ability to disrupt biofilms and prevent their formation, as described above, led us to investigate cysteamine’s potential as a mucolytic/mucus penetration-enhancing agent. Specifically, the impact of cysteamine on the macrorheology (viscosity and elasticity) of mucin and DNA [22-24] was assessed. Mucin and DNA are important macromolecular components of cystic fibrosis sputum [24]. The effect of cysteamine on mucin and DNA viscoelasticity was assessed and compared to mucoactive agents already used in cystic fibrosis therapy (N-Acetylcysteine and rhDNAse I) or under investigation as potential therapeutic agents for cystic fibrosis (alginate lysae) [22,23]. The increased velocity (reduced viscosity and elasticity) observed for mucin (Figure 3) following a single exposure to cysteamine (8.8 mm/sec ±0.5) or cysteamine hydrochloride (8.4 mm/sec ±0.5) versus untreated mucin (<1 mm/sec) indicates significant mucoactive potential. Moreover, the impact of cysteamine/cysteamine hydrochloride on mucin macrorheology was greater than that observed for N-acetylcysteine (N-acetylcysteine treated mucin having a velocity of 4.4 mm/sec ±1.0), DNase I and alginate lyase (neither of these mucoactive agents having any impact on mucin velocity/rheology properties). The effect of cysteamine on changes in DNA viscosity (Figure 4) was less pronounced than with mucin and effectively neutral. Unsurprisingly, cysteamine was inferior to rhDNase I.

### Effect of cysteamine on mucin production by normal human bronchial epithelial (NHBE) cells

**Figure 5:**
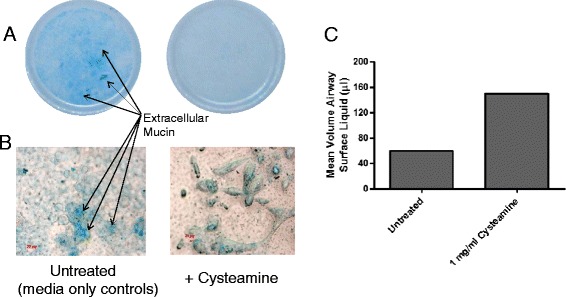
**Cysteamine disrupts production of normal human bronchial epithelial (NHBE) cell-derived mucus.** Differentiated NHBE monolayers were exposed (basally) to 1 mg/ml cysteamine or control culture media for 7 days. Mucin production at the apical aspect was then assessed by Alcian blue staining macro- and microscopically (panels **A** & **B**) and the amount of free, non-mucin bound airway surface fluid quantified (panel **C**).

Although the isolated porcine mucin and DNA rheology assays are very good indicators of cysteamine’s mucoactive potential, we went further to assess its properties against mucin as a component of respiratory epithelial cell-derived mucus; in a more complex and physiological relevant human system employing differentiated NHBE cells. Figure 5(A) demonstrates the presence of mucin within NHBE monolayer cultures as a function of the intensity of Alcian blue staining and that it is significantly reduced (less blue, only background staining versus specific matter) on monolayers exposed for 7 d to cysteamine versus those exposed to control media only. Figure 5(B) demonstrates the presence at a microscopic level of more Alcian blue-stained material within the cultures in control non-cysteamine treated cells versus cysteamine-treated cells. Finally, Figure 5(C) demonstrates that the disruption/reduction of mucin in the cysteamine treated cultures as indicated by less Alcian Blue staining also results in a greater volume of free apical surface liquid/fluid. This is to be expected if less intact mucin molecules are present to bind water and other airway surface fluid components in the apical mucus layer. The NHBE cell assay confirms the mucoactive properties of cysteamine in a physiologically relevant primary lung epithelial monolayer based system and also demonstrated that even relatively high levels of cysteamine were non-toxic to the NHBE cells.

### Antimicrobial activity of Cysteamine

Following the identification of antibiofilm properties that also pointed to a direct antimicrobial activity of cysteamine against *P. aeruginosa* PAO1, ATCC27853 and DSMZ1299, the antibacterial potential of cysteamine was assessed against a panel of CF bacterial pathogens. Table 1 shows a summary of the direct antimicrobial activity (median MIC_100_ and range (μg/ml)) of cysteamine against a range of Gram negative and Gram positive bacteria characteristic of CF pulmonary infections, including clinical isolates and mucoid and non-mucoid *P. aeruginosa*. Mucoid strains are alginate producers and are therefore generally less susceptible to conventional antibiotics and (non-alginate lyase) mucoactive agents. A complete list of individual MICs for all strains tested can be found in Additional file 1: Table S1. Cysteamine showed direct antimicrobial activity (MIC_100_) at concentrations in the range of 250–500 μg/ml against *P. aeruginosa* and other CF pathogens including *Burkholderia cepacia* complex, *Staphylococcus aureus* and also emerging pathogens including *Achromobacter xylosidans*, *Stenotrophomonas* spp and *Ralsatonia* spp (data not shown)*.*

**Figure 6:**
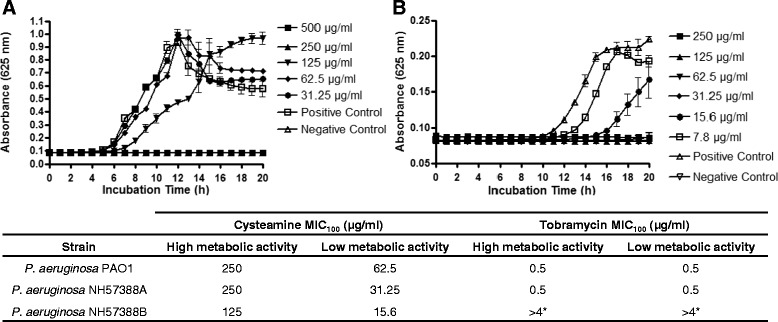
**Antibacterial efficacy of cysteamine and tobramycin on** ***P. aeruginosa*** **incubated under nutrient-limiting and Nutrient replete conditions.** The MIC_100_ of *P. aeruginosa* PAO1, *P. aeruginosa* NH57388A and *P. aeruginosa* NH57388B were determined according to standard CLSI conditions for – **A)** nutrient replete media (MH broth), whereas – **B)** M9 minimal medium was substituted for MH broth to provide nutrient-limiting conditions. The graphs **(A & B)** show data for *P. aeruginosa* PAO1. The table shows the mean MIC of triplicate samples from triplicate experiments. Error bars represent the Standard Error of the Mean. *P. aeruginosa* NH57388A is a mucoid strain. *P. aeruginosa* NH57388B is a non-mucoid strain. * - *P. aeruginosa* NH57388B is tobramycin resistant.

Bacteria in the CF lung grow in biofilms under nutrient-limited conditions [25], so the antibacterial efficacy of cysteamine was assessed under nutrient limiting (M9 minimal medium) and excess nutrient (MH broth) conditions. Under the more physiologically relevant, nutrient-limiting assay conditions (Figure 6), the MIC_100_ of cysteamine against *P. aeruginosa* was reduced by 4–8 fold, whereas the MIC_100_ of tobramycin was unchanged, suggesting a cysteamine mechanism of action that is not dependent on metabolic activity. This data suggests that not only is cysteamine a biofilm prevention and disrupting agent (Figures 1 and 2), but is also directly antibacterial against CF pathogens (Table 1) and importantly, demonstrates improved efficacy under nutrient limited conditions such as within the CF lung environment.

### Antimicrobial activity of cysteamine in combination with conventional cystic fibrosis antibiotics

As an antibiofilm/mucolytic agent, cysteamine would almost certainly be used in a clinical setting alongside currently available antibiotics as a means to facilitate their access to target pathogens. A synergistic relationship between tobramycin and cysteamine was observed in our biofilm assays (as described above; Figures 1 & 2), so we next investigated the antibacterial potential of cysteamine in combination with CF antibiotics (Table 2). Indeed, at least additive or synergistic activity was observed in combination with tobramycin, ciprofloxacin, colistin and gentamicin against five strains of *P. aeruginosa* (PAO1, PA14, Pa058, NH57388D and NH57388A). Most significantly, the MIC_100_ of colistin, tobramycin and gentamicin were reduced up to 16-fold when combined with cysteamine. In certain cases, antibiotic resistance was reversed. For example, *P. aeruginosa* Pa058 is a ciprofloxacin resistant clinical isolate (MIC_100_ = 32 μg/ml), based on CLSI breakpoints [26] and has a cysteamine MIC_100_ of 500 μg/ml. However, when used in combination an additive FIC was obtained and the MICs of cysteamine and ciprofloxacin were 250 μg/ml and 1 μg/ml, respectively. This means that in the presence of cysteamine, *P. aeruginosa* Pa058 becomes sensitive to ciprofloxacin, i.e. reversal of resistance. In another instance, the tobramycin resistant strain, *P. aeruginosa* NH57388D (MIC_100_ = 16 μg/ml) had its resistance reversed in the presence of cysteamine (2 μg/ml). Because aminoglycosides are associated with a degree of nephrotoxicity and ototoxicity [27], their use in combination with cysteamine may provide a means to lower the antibiotic concentration required while retaining or even enhancing their antimicrobial effect whilst reducing toxicity. Furthermore, the combined use of antimicrobials with different mechanisms of action is believed to aid in the ever-increasing incidence of antibiotic resistance [28].

**Table 2:** **Analysis of the Fractional Inhibitory Concentration Index (FICI) of Cysteamine with Cystic Fibrosis-relevant Antibiotics versus** ***P. aeruginosa***

	**Tobramycin**			**Ciprofloxacin**			**Colistin**			**Gentamicin**		
**Strain**	**Syn**	**Add**	**Ind**	**Syn**	**Add**	**Ind**	**Syn**	**Add**	**Ind**	**Syn**	**Add**	**Ind**
**PAO1**	6	5	0	0	3	5	3	4	4	1	5	0
**Pa14**	0	7	0	0	2	5	5	7	0	0	5	0
**Pa058**	0	8	0	0	11	1	0	6	4	0	6	0
**57388A**	2	5	0	0	2	1	2	6	0	1	6	0
**57388D**	4	4	0	0	4	1	2	7	0	1	5	0

Numbers in the table indicate the number of replicates demonstrating these effects. Syn = synergy, Add = Additive, Ind = Indifferent. No antagonism was observed for any of the cysteamine-antibiotic combinations. *P. aeruginosa* Pa14 is colistin resistant, *P. aeruginosa* Pa058 is ciprofloxacin resistant, *P. aeruginosa* 57388A is colistin and tobramycin resistant and *P. aeruginosa* 57388A is ciprofloxacin and tobramycin resistant. *P. aeruginosa* PAO1 is sensitive to all antibiotics tested.

The activity of cysteamine in combination with tobramycin was next determined under more physiologically relevant conditions that better resemble cystic fibrosis sputum; the biological matrix in which cysteamine will need to function if it is to be effective *in vivo* [19]. The MIC of cysteamine against 4 *P. aeruginosa* (PAO1, ATCC27853, DSMZ1128 and DSMZ1299) in artificial sputum medium (Table 3) ranged between 250 and 500 μg/ml, as it did under standard CLSI conditions (MH broth). In addition to there being no significant inhibition of cysteamine antimicrobial activity under artificial sputum conditions, the same degree of synergy between tobramycin and cysteamine was observed as per standard CLSI broth microdilution conditions (Table 3), the FIC index for the 4 *P. aeruginosa* strains was between 0.5 and 1 in both conditions. Of note, the CLSI interpretive standard for tobramycin sensitivity against *P. aeruginosa* is ≤4 μg/ml, emphasising the dramatic reduction in MIC in the presence of cysteamine in all conditions in which we tested its activity.

**Table 3:** **Comparison of the Antibacterial Activity of Cysteamine and Tobramycin in Artificial Sputum Medium and under standard CLSI conditions**

	**Mueller Hinton Broth MIC**_**100**_ **(μg/ml)**		**Artificial Sputum Medium MIC**_**100**_ **(μg/ml)**	
**Isolate**	**Cysteamine**	**Tobramycin**	**Cysteamine**	**Tobramycin**
*P. aeruginosa* PAO1	250	1	500	4
*P. aeruginosa* DSMZ1128	250	1	250	2
*P. aeruginosa* DSMZ1299	250	1	250	1
*P. aeruginosa* ATCC27853	250	1	500	2

Data represents the mean MIC_100_ (μg/ml) of triplicate samples from triplicate experiments. MICs were determined using the broth microdilution procedure from CLSI Approved Standard M07-A9 [18], with artificial sputum medium substituted for MH broth as appropriate. Data represents the mean MIC_100_ (μg/ml) of triplicate samples from triplicate experiments.

**Figure 7:**
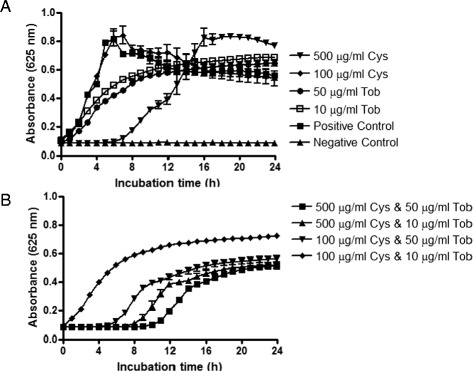
**Post-Antimicrobial effect (PAE) of cysteamine, tobramycin and combinations thereof.** The impact of cysteamine or tobramycin **(A)** and combinations thereof **(B)** on the recovery of growth of *P. aeruginosa* PAO1 cells that had been exposed to either/both of these antimicrobial agents for 16 h was monitored for 24 h post termination of cysteamine or/and tobramycin treatment at 37°C in a BioTek Synergy HT microplate reader.

Following biofilm prevention experiments, samples were analysed to determine whether cysteamine elicited a post-antimicrobial effect (PAE) on bacteria. Kinetic growth assays on these samples did reveal a post-antimicrobial effect; namely in that delayed growth of bacteria from cysteamine-treated (non-biofilm forming) cells was observed, but interestingly, no delayed growth/PAE was observed in samples from tobramycin-treated cells/biofilms (Figure 7A). Combinations of tobramycin and cysteamine however exerted a greater PAE than cysteamine administered as a mono-treatment (Figure 7B). It is likely that the PAE seen after treatment with combinations of these antimicrobial agents is a result of continued/sustained interactions with their bacterial targets. For example, the PAE of rifampicin, which targets bacterial RNA polymerase, correlates with the time taken for recovery of RNA and protein synthesis [29]. The PAE observed for cysteamine potentially has significant implications for clinical dosing regimens; a combination of cysteamine and tobramycin may have the potential for longer dosing intervals and lower doses therein than treatment with tobramycin alone.

### *In vivo* efficacy & tolerability

**Figure 8:**
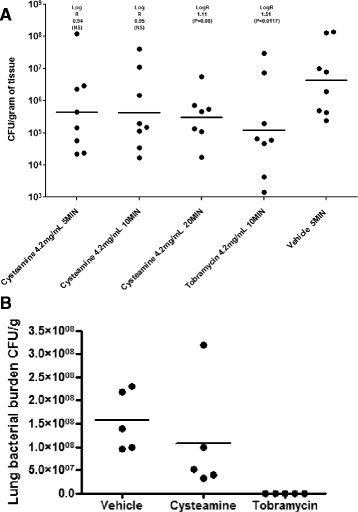
**Antimicrobial activity of cysteamine following nebulisation (A) and intra-tracheal dosing (B) in a mouse acute lung infection model. (A)** The clinical strain *P. aeruginosa* EUPPA103 was administered at ~6.5 × 10^4^ CFU/mouse by intranasal injection under temporary inhaled anesthesia. Mice were placed within sealed nebulisation chamber and exposed to cysteamine at 4.2 mg/ml for 5, 10 or 20 minutes (total 1 dose) or tobramycin at 4.2 mg/ml in aqueous solution for 10 minutes via aerosol delivery system 1 hour post-infection. Experimental endpoint was lung tissue burden 25 h post-infection. Vehicle was sterile physiological water. The lower limit of detection was approximately 50 cfu/g of tissue. **(B)** The clinical strain *P. aeruginosa* ATCC27853 was administered at 3 × 10^4^ - 1 × 10^5^ cfu/40 μl/mouse by intranasal injection under temporary inhaled anesthesia. Mice were given two doses (5 mg/kg each) of cysteamine or tobramycin delivered intratracheally 10 min and 6 h after infection. Experimental endpoint was lung tissue burden 26 h post-infection. Vehicle was sterile physiological water. The lower limit of detection was approximately 50 cfu/g of tissue.

To assess whether the *in vitro* antibiofilm and antibacterial data derived in our studies translated in a more clinically relevant, complex biological setting, we employed a well-established mouse model of *P. aeruginosa* lung infection and determined the impact therein of cysteamine exposure on lung bacterial burdens. We also assessed how well tolerated cysteamine was when delivered via the respiratory route as to date, its safety and toxicological potential have not been determined by this mode of application. Cysteamine was well tolerated and did not cause any adverse effects when administered to mice via two respiratory routes; either by a single dose of cysteamine or tobramycin delivered by nebulisation (4.2 mg/ml for 5 min, 10 min or 20 min) following infection with *P. aeruginosa* EUPPA103 (Figure 8A), or two doses (5 mg/kg each) delivered intratracheally 10 min and 6 h after infection with *P. aeruginosa* ATCC27853 (Figure 8B). These experiments provide the first evidence of *in vivo* antibacterial efficacy of cysteamine in respiratory tissue. In the mice infected with *P. aeruginosa* EUPPA103, nebulised cysteamine (4.2 mg/ml, 5 – 20 min exposure) significantly reduced bacterial lung burden (LogR 0.94-1.11 cfu/g; P ≤ 0.001) compared to vehicle only treated mice (StatsDirect Kruskal-Wallis test: Squared ranks approximate equality of variance test). Tobramycin (4.2 mg/ml aqueous solution) treated mice also demonstrated statistically significant reduction in lung burden (LogR 1.51 CFU/g; P = 0.0 117) (StatsDirect Kruskal-Wallis test: all pairwise comparisons (Conover-Inman)). A trend to dose dependency for cysteamine was observed, with greatest efficacy demonstrated by 20 min exposure (5.48 log_10_ CFU/g) compared with 5 min (5.65 log_10_ CFU/g) and 10 min exposure (5.64 log_10_ CFU/g). In the mice infected with *P. aeruginosa* ATCC27853, intratracheal administration of cysteamine reduced bacterial burden in the lungs from 8.17 ± 0.08 log_10_ CFU/g (control animals) to 7.86 ± 0.18 log_10_ CFU/g (cysteamine-treated animals).

It is worth noting that these *in vivo* assays employed only one or two doses of cysteamine, whereas it is most likely that multiple doses (i.e. daily or twice daily dosing) would be expected for the treatment of CF in a clinical setting. It is therefore not unreasonable to assume at least an equal, or even greater, reduction in respiratory tract bacterial burden following repeat dosing with cysteamine, especially considering its sustained *in vitro* post-antimicrobial effect. Furthermore, nebulisation may not be the administration route via which cysteamine/Lynovex® is delivered; dry powder inhalation and other modes also being applicable and potentially providing greater drug delivery efficiency.

## Conclusions

As a first-in-class combination mucolytic-antibiofilm-antimicrobial agent, cysteamine (as Lynovex®) has the potential to offer a novel way forward in the treatment of the chronic and recurrent respiratory infections of cystic fibrosis. Previous clinical experience and our own data point to cysteamine as being safe. Furthermore, cysteamine should be applicable in all cystic fibrosis disease genotypes/phenotypes, not a mutation-specific treatment in its recovery/improvement of respiratory health and function.

Cysteamine could potentially break the cycle of progressive lung damage and recurrent infections in cystic fibrosis. Cysteamine’s manifold actions of mucus and biofilm disruption and antimicrobial impact on the bacteria these structures support could achieve this end. As an adjunct therapy with existing antibiotic treatment regimens, a further advantage of cysteamine/Lynovex® is that its use would not require significant modification to cystic fibrosis therapy schedules already in place. Instead, cysteamine/Lynovex® could potentiate and extend the effects of the ‘older’ therapeutics that remain the core of existing exacerbation interventions and longer term maintenance.

Lynovex® (cysteamine) is already designated as an orphan therapeutic candidate for the treatment of cystic fibrosis and our intention is to move forward with development of this compound in oral and inhaled form for the control/eradication of the CF respiratory microbial burden. This would apply both to exacerbations and also longer term maintenance of pulmonary health in adult and paediatric cystic fibrosis patients.

## Additional file

Additional file 1: Table S1. MIC of Cysteamine and N-Acetylcysteine Against Representative Type Strains and Clinical Isolates of Cystic Fibrosis Pathogens. Data shown are median values of triplicates samples from triplicate experiments; MIC_100,_ MIC_90_ and MIC_50_: concentration inhibiting 100%, 90% and 50% bacterial growth, respectively. P = Pseudomonas, B = Burkholderia, A = Acinetobacter, Al = Alcaligenes, Ch = Chryseomonas, E = Escherichia, K = Klebsiella, C = Clostridium, S = Staphylococcus.
