# Supplementary material for: Cysteamine (Lynovex®), a novel mucoactive antimicrobial & antibiofilm agent for the treatment of cystic fibrosis
Source: Orphanet J Rare Dis. 2014 Nov 30;9:189. doi: 10.1186/s13023-014-0189-2 (PMC4260250; doi:10.1186/s13023-014-0189-2)
Supplement: Additional file 1: Table S1. — MIC of Cysteamine and N-Acetylcysteine Against Representative Type Strains and Clinical Isolates of Cystic Fibrosis Pathogens. Data shown are median values of triplicates samples from triplicate experiments; MIC100, MIC90 and MIC50: concentration inhibiting 100%, 90% and 50% bacterial growth, respectively. P = Pseudomonas, B = Burkholderia, A = Acinetobacter, Al = Alcaligenes, Ch = Chryseomonas, E = Escherichia, K = Klebsiella, C = Clostridium, S = Staphylococcus. [file 13023_2014_189_MOESM1_ESM.doc]

**Table S1: MIC of Cysteamine and N-Acetylcysteine Against Representative Type Strains and Clinical Isolates of Cystic Fibrosis Pathogens.** Data shown are median values of triplicates samples from triplicate experiments; MIC_100_, MIC_90_ and MIC_50_: concentration inhibiting 100%, 90% and 50% bacterial growth, respectively. P = Pseudomonas, B = Burkholderia, , A = Acinetobacter, Al = Alcaligenes, Ch = Chryseomonas, E = Escherichia, K = Klebsiella, C = Clostridium, S = Staphylococcus.

|  |  |  | **Cysteamine concentration (µg/ml)** | | |
| --- | --- | --- | --- | --- | --- |
| **Genus & Species** | **Code** | **ORIGIN** | **MIC_100_** | **MIC_90_** | **MIC_50_** |
| ***P. aeruginosa*** | **DSMZ1128** | Otitis externa | 250 | 250 | 250 |
| ***P. aeruginosa*** | **DSMZ1299** | Sputum | 500 | 250 | 250 |
| ***P. aeruginosa*** | **PAO1 (BAA-47)** | ND | 250 | 250 | 250 |
| ***P. aeruginosa*** | **ATCC27853** | Blood | 250 | ND | ND |
| ***P. aeruginosa*** | **NH57388A** | CF sputum | 250 | 250 | 250 |
| ***P. aeruginosa*** | **NH57388B** | Hoffmann et al., 2005 | 250 | 250 | 125 |
| ***P. aeruginosa*** | **NH57388C** | Hoffmann et al., 2005 | 250 | 250 | 250 |
| ***P. aeruginosa*** | **NH57388D** | Hoffmann et al., 2005 | 250 | 250 | 250 |
| ***P. aeruginosa*** | **DSMZ50071** | Type Strain | 500 | 500 | 250 |
| ***P. aeruginosa*** | **UCBPP-PA14** | Burn | 500 | 500 | 250 |
| ***P. aeruginosa*** | **Pa058** | ND | 500 | 500 | 250 |
| ***P. aeruginosa*** | **Pa492a** | ND | 250 | 125-250 | 125 |
| ***P. aeruginosa*** | **PA0579** | ND | 250 | 125-250 | 125-250 |
| ***P. aeruginosa*** | **04-51393** | Clinical isolate | 250 | 250 | 250 |
| ***P. aeruginosa*** | **04-50400** | Clinical isolate | 250 | 250 | 250 |
| ***P. aeruginosa*** | **04-44722** | Clinical isolate | 250 | 250 | 250 |
| ***P. aeruginosa*** | **04-33171** | Clinical isolate | 250 | 250 | 250 |
| ***P. aeruginosa*** | **04-28150** | Clinical isolate | 250 | 250 | 250 |
| ***P. aeruginosa*** | **03-77249** | Clinical isolate | 250 | 250 | 250 |
| ***P. aeruginosa*** | **03-19100A** | Clinical isolate | 500 | 500 | 250 |
| ***P. aeruginosa*** | **04-53541** | Clinical isolate | 500 | 500 | 250 |
| ***P. aeruginosa*** | **04-56216B** | Clinical isolate | 250 | 250 | 250 |
| ***P. aeruginosa*** | **04-66124A** | Clinical isolate | 250 | 250 | 125 |
| ***P. aeruginosa*** | **04-77538** | Clinical isolate | 250 | 250 | 250 |
| ***P. aeruginosa*** | **04-82739B** | Clinical isolate | 250 | 250 | 250 |
| ***P. aeruginosa*** | **05-34737A** | Clinical isolate | 250 | 250 | 250 |
| ***P. aeruginosa*** | **09-7037A** | Clinical isolate | 250 | 250 | 250 |
| ***P. aeruginosa*** | **09-12898A** | Clinical isolate | 250 | 250 | 250 |
| ***P. aeruginosa*** | **09-13940A** | Clinical isolate | 500 | 500 | 250 |
| ***P. aeruginosa*** | **09-20407B** | Clinical isolate | 500 | 500 | 250 |
| ***P. aeruginosa*** | **09-28588** | Clinical isolate | 250 | 250 | 125 |
| ***P. aeruginosa*** | **09-24075A** | Clinical isolate | 250 | 250 | 250 |
| ***P. aeruginosa*** | **09-33354B** | Clinical isolate | 250 | 250 | 250 |
| ***P. aeruginosa*** | **09-33899** | Clinical isolate | 250 | 250 | 250 |
| ***P. aeruginosa*** | **09-36021** | Clinical isolate | 250 | 250 | 250 |
| ***P. aeruginosa*** | **09-58387B** | Clinical isolate | 250 | 250 | 125 |
| ***P. aeruginosa*** | **09-58931** | Clinical isolate | 250 | 250 | 250 |
| ***P. aeruginosa*** | **09-59259** | Clinical isolate | 250 | 250 | 125 |
| ***P. aeruginosa*** | **09-59270** | Clinical isolate | 250 | 250 | 125 |
| ***P. aeruginosa*** | **09-59498** | Clinical isolate | 250 | 250 | 125 |
| ***P. aeruginosa*** | **09-59631A** | Clinical isolate | 250 | 250 | 250 |
| ***P. fluorescens*** | **CFSYN_975** | CF sputum | 500 | 250 | 250 |
| ***P. fluorescens*** | **CFSYN_1036** | CF sputum | 250 | 250 | 125 |
| ***P. putida*** | **CFSYN_817** | CF sputum | 250 | 250 | 250 |
| ***B. cenocepacia*** | **CFSYN_936** | CF sputum | 250 | 250 | 125 |
| ***B. cenocepacia*** | **CFSYN_1045** | CF sputum | 500 | 500 | 250 |
| ***B. cenocepacia* (ET12)** | **CFSYN_1112** | CF sputum | 250 | 250 | 125 |
| ***B. cepacia*** | **CFSYN_946** | CF sputum | 1000 | 500 | 250 |
| ***B. cepacia*** | **NCTC10743** | ND | 500 | 250 | 125 |
| ***B. cepacia*** | **NCTC10744** | Bronchial washings | 500 | 250 | 125 |
| ***B. multivorans*** | **CFSYN_945** | CF sputum | 500 | 250 | 62.5 |
| ***B.multivorans*** | **CFSYN_954** | CF sputum | 500 | 500 | 250 |
| ***B. multivorans*** | **CFSYN_1081** | CF sputum | 500 | 500 | 250 |
| ***A. baumanii*** | **NCTC12156** | Type Strain | >250 | >250 | 250 |
| ***A. calcoaceticus*** | **NCTC7422** | Burn | >250 | 125 | 125 |
| ***A. faecalis*** | **CFSYN_503** | CF sputum | 500 | 500 | 250 |
| ***A. faecalis*** | **CFSYN_699** | CF sputum | 250 | 250 | 125 |
| ***Ch. indologenes*** | **CFSYN_819** | CF sputum | 500 | 500 | 250 |
| ***Ch. meningosepticum*** | **CFSYN_535** | CF sputum | 250 | 250 | 62.5 |
| ***E. coli*** | **NCTC9434** | Vagina | 250 | 250 | 15.6 |
| ***E. coli*** | **NCTC1093** | Faeces | 500 | 250 | 7.8 |
| ***E. coli*** | **NCTC9001** | Urine, cystitis | 250 | 250 | 62.5 |
| ***K. pneumonia*** | **NCTC8849** | Respiratory tract | 250 | 250 | 31.25 |
| ***K. pneumonia*** | **NCTC5056** | Pneumonia | 1000 | 1000 | 125 |
| ***K. pneumonia*** | **NCTC9633** | Type Strain | 250 | 250 | 125 |
| ***C. difficile*** | **NCTC11209** | Type Strain | 1000 | 1000 | 1000 |
| ***S. epidermidis*** | **ATCC12228** | FDA Type Strain | 500 | 500 | 250 |
| ***S. epidermidis*** | **ATCC35984** | Catheter sepsis | 500 | 500 | 125 |
| ***S. aureus*** | **ATCC25923** | ND | 500 | 500 | 500 |
| ***S. aureus*** | **DSMZ11729** | Blood; MRSA | 500 | 500 | 500 |
| ***S. aureus*** | **NCTC12493** | MRSA control strain | 500 | 500 | 125 |
| ***S. aureus*** | **BAA-1717** | Severe sepsis | 500 | ND | 15.6 |
| ***S.aureus*** | **SACF660** | CF sputum | 500 | 250 | 250 |
| ***S.aureus*** | **SACF661** | CF sputum | 500 | 500 | 250 |
| ***S.aureus*** | **SACF663** | CF sputum | 250 | 250 | 250 |
| ***S.aureus*** | **SACF665** | CF sputum | 500 | 250 | 125 |
| ***S.aureus*** | **SACF666** | CF sputum | 250 | 250 | 250 |
| ***S.aureus*** | **SACF636** | CF sputum; MRSA | 250 | 250 | 250 |
| ***S.aureus*** | **SACF642** | CF sputum; MRSA | 250 | 250 | 250 |
| ***S.aureus*** | **SACF652** | CF sputum; MRSA | 250 | 250 | 250 |
| ***S.aureus*** | **SACF662** | CF sputum; MRSA | 250 | 250 | 250 |
| ***S.aureus*** | **SACF667** | CF sputum; MRSA | 250 | 250 | 125 |
